# Cilofexor in Patients With Compensated Cirrhosis Due to Primary Sclerosing Cholangitis: An Open-Label Phase 1B Study

**DOI:** 10.14309/ctg.0000000000000744

**Published:** 2024-07-01

**Authors:** Cynthia Levy, Stephen Caldwell, Parvez Mantry, Velimir Luketic, Charles S. Landis, Jonathan Huang, Edward Mena, Rahul Maheshwari, Kevin Rank, Jun Xu, Vladislav A. Malkov, Andrew N. Billin, Xiangyu Liu, Xiaomin Lu, William T. Barchuk, Timothy R. Watkins, Chuhan Chung, Robert P. Myers, Kris V. Kowdley

**Affiliations:** 1 Division of Digestive Health and Liver Diseases, University of Miami Miller School of Medicine, Miami, Florida, USA;; 2 Schiff Center for Liver Diseases, University of Miami, Miami, Florida, USA;; 3 University of Virginia School of Medicine, Charlottesville, Virginia, USA;; 4 Methodist Transplant Specialists, Dallas, Texas, USA;; 5 Virginia Commonwealth University School of Medicine, Richmond, Virginia, USA;; 6 Univerisity of Washington School of Medicine, Seattle, Washington, USA;; 7 University of Rochester School of Medicine and Dentistry, Rochester, New York, USA;; 8 Pasadena Liver Center, Pasadena, California, USA;; 9 Piedmont Transplant Institute, Atlanta, Georgia, USA;; 10 MNGI Digestive Health, Minneapolis, Minnesota, USA;; 11 Gilead Sciences, Inc., Foster City, California, USA;; 12 Liver Institute Northwest, Seattle, Washington, USA.

**Keywords:** cilofexor, compensated cirrhosis, efficacy, primary sclerosing cholangitis, safety

## Abstract

**INTRODUCTION:**

This proof-of-concept, open-label phase 1b study evaluated the safety and efficacy of cilofexor, a potent selective farnesoid X receptor agonist, in patients with compensated cirrhosis due to primary sclerosing cholangitis.

**METHODS:**

Escalating doses of cilofexor (30 mg [weeks 1–4], 60 mg [weeks 5–8], 100 mg [weeks 9–12]) were administered orally once daily over 12 weeks. The primary endpoint was safety. Exploratory measures included cholestasis and fibrosis markers and pharmacodynamic biomarkers of bile acid homeostasis.

**RESULTS:**

Eleven patients were enrolled (median age: 48 years; 55% men). The most common treatment-emergent adverse events (TEAEs) were pruritus (8/11 [72.7%]), fatigue, headache, nausea, and upper respiratory tract infection (2/11 [18.2%] each). Seven patients experienced a pruritus TEAE (one grade 3) considered drug-related. One patient temporarily discontinued cilofexor owing to peripheral edema. There were no deaths, serious TEAEs, or TEAEs leading to permanent discontinuation. Median changes (interquartile ranges) from baseline to week 12 (predose, fasting) were −24.8% (−35.7 to −7.4) for alanine transaminase, −13.0% (−21.9 to −8.6) for alkaline phosphatase, −43.5% (−52.1 to −30.8) for γ-glutamyl transferase, −12.7% (−25.0 to 0.0) for total bilirubin, and −21.2% (−40.0 to 0.0) for direct bilirubin. Least-squares mean percentage change (95% confidence interval) from baseline to week 12 at trough was −55.3% (−70.8 to −31.6) for C4 and −60.5% (−81.8 to −14.2) for cholic acid. Fasting fibroblast growth factor 19 levels transiently increased after cilofexor administration.

**DISCUSSION:**

Escalating doses of cilofexor over 12 weeks were well tolerated and improved cholestasis markers in patients with compensated cirrhosis due to primary sclerosing cholangitis (NCT04060147).

## INTRODUCTION

Primary sclerosing cholangitis (PSC) is a rare chronic liver disease, affecting fewer than 200,000 individuals in the United States (1). PSC is characterized by inflammation, concentric fibrosis, and multifocal strictures of the bile ducts (1,2). Although PSC can be asymptomatic, many patients experience pruritus, fatigue, ascending cholangitis, and poor health-related quality of life (HRQoL) (3–5). Although the pathogenesis of PSC is unknown, there is a close association with inflammatory bowel disease (IBD) (1,6). PSC is also associated with an increased risk of hepatobiliary and gastrointestinal cancers, particularly cholangiocarcinoma (7,8). PSC is progressive, whereby continued hepatic inflammation in compensated cirrhosis can eventually lead to decompensated cirrhosis, portal hypertension, and hepatic failure (1,2). There are no proven medical interventions that delay the progression of PSC and compensated cirrhosis. Effective pharmacological therapy to halt disease progression therefore remains an unmet need.

The farnesoid X receptor (FXR) is a major regulator of bile acid homeostasis and is therefore a potential therapeutic target for PSC (9). Activation of FXR in the intestine stimulates the release of fibroblast growth factor 19 (FGF19), a key hormonal regulator of postprandial metabolism that inhibits bile acid synthesis (9). In the liver, activation of FXR and release of FGF19 inhibits the expression of cholesterol 7α-hydroxylase, the rate-limiting enzyme in bile acid synthesis and excretion (10,11). This results in reductions of 7a-OH-cholesterol and the subsequent metabolite 7a-OH-4-cholesterol-3-one (C4). Activation of FXR also inhibits sterol 12α-hydroxylase, which together with cholesterol 7α-hydroxylase, prevents the synthesis of primary bile acids such as cholic acid from C4 (12).

Cilofexor is an orally available, potent, and selective FXR agonist that may have a potential therapeutic benefit in patients with PSC. A 12-week, randomized, double-blind, placebo-controlled phase 2 study (NCT02943460) showed that cilofexor 30 mg and 100 mg improved markers of cholestasis and liver injury, was well tolerated, and did not exacerbate pruritus (a common side effect of FXR agonists) in patients with PSC without cirrhosis (13). The cilofexor 100 mg dose provided greater therapeutic benefit than the cilofexor 30 mg dose (13). The sustained safety and efficacy of cilofexor was subsequently demonstrated in a 96-week open-label extension study (14). The phase 3 PRIMIS study investigating the effects of cilofexor on progression of liver fibrosis in patients with PSC without cirrhosis (NCT03890120) (15) was recently terminated for futility; however, no new safety issues were identified. The efficacy of cilofexor in patients with cirrhosis, those with the largest unmet need, remains unclear.

To understand the safety, tolerability, and efficacy of cilofexor in patients with cirrhotic PSC, a proof-of concept, phase 1b study was designed to include patients with PSC and compensated cirrhosis (NCT04060147). Only patients who met clinical, histologic, or noninvasive criteria consistent with cirrhosis and who had preserved liver function were included in the study. The study design was informed by a previous phase 1, open-label, single-dose pharmacokinetic trial, which showed a two-fold higher plasma exposure to cilofexor in patients with impaired hepatic function vs those with preserved hepatic function (16). The increase in plasma exposure was attributed to impaired hepatic organic anion transporting polypeptide uptake and reduced metabolic clearance by hepatic cytochrome P450 (CYP) 2C8 and 3A4 (16). Because the magnitude of these effects was similar (i.e., the reduction in organic anion transporting polypeptide protein levels was similar to the reduction in CYP protein levels), their overall impact on hepatic exposure to cilofexor is expected to be neutral (16). Thus, a 100 mg dose was still anticipated to be required for full efficacy in patients with PSC and compensated cirrhosis. Therefore, this phase 1b study was designed to assess the safety, tolerability, and efficacy of escalating doses of cilofexor (30, 60, and 100 mg) in patients with PSC and compensated cirrhosis.

Here, we report the safety and efficacy results from this phase 1b open-label study including the effects of cilofexor on markers of cholestasis and fibrosis and on pharmacodynamic (PD) biomarkers of bile acid homeostasis.

## METHODS

### Study design and treatment

This was a proof-of-concept, open-label phase 1b study that evaluated the safety, tolerability, and efficacy of cilofexor in patients with PSC and compensated cirrhosis. The study took place across 8 centers in the United States and consisted of a 4-week screening period, a 12-week treatment period, and a follow-up visit 4 weeks later. Participants who met enrollment eligibility criteria received treatment with escalating doses of cilofexor: 30 mg once daily (QD; weeks 1–4), 60 mg QD (weeks 5–8), and 100 mg QD (weeks 9–12). Dose-escalation decisions were made by the principal investigators (PIs) based on their evaluations of patients' tolerance to the study drug. Dosing could be interrupted by the PI for up to 4 consecutive weeks if a patient experienced an adverse event considered to be related to the study drug. Compliance was measured by counting patients' remaining tablets at each visit.

The development or worsening of pruritus during the study could be managed by nonpharmacologic interventions such as skin moisturization, minimized heat exposure, and the avoidance of skin irritants; topical corticosteroids; oral antihistamines; or bile acid sequestrants such as cholestyramine. Bile acid sequestrants had to be taken more than 4 hours before or after cilofexor dosing. A formal management plan was devised for patients who developed significant pruritus, which included temporary cilofexor dose interruption, dose reduction, and supportive management with antihistamines and bile acid sequestrants.

The study protocol was approved by the institutional review board at all participating centers. The study was conducted in accordance with the Declaration of Helsinki and relevant local regulations. All patients provided written informed consent.

### Study population

Men and women aged 18–70 years with PSC and compensated cirrhosis were enrolled in the study. PSC was diagnosed by cholangiogram or liver biopsy, and compensated cirrhosis was diagnosed if patients met at least one of the following criteria: historical liver biopsy that revealed Ludwig stage F4 fibrosis (or equivalent), abdominal imaging with features consistent with cirrhosis, and/or liver stiffness by FibroScan (Echosens SA, Paris, France) ≥14.4 kPa (17), Enhanced Liver Fibrosis test (ELF; Siemens Healthcare GmbH, Erlangen, Germany) ≥11.3 (18), or FibroTest (BioPredictive S.A.S., Paris, France) ≥0.75 (19). Eligible patients had an estimated glomerular filtration rate of above 60 mL/min, an alanine aminotransferase (ALT) level of ≤5 × the upper limit of normal, a total bilirubin level of ≤2 mg/dL (unless the patient was known to have Gilbert syndrome or hemolytic anemia), an international normalized ratio (INR) of ≤1.4 (unless due to therapeutic anticoagulation), and a platelet count of ≥ 75,000/μL (excluding patients with high-risk esophageal or gastric varices). For patients receiving ursodeoxycholic acid (UDCA) therapy, the dose of UDCA must have been stable (in the opinion of the PI) for at least 6 months before screening. For patients not on UDCA therapy, no UDCA use was allowed for at least 6 months before screening.

Patients were excluded from the study if they were pregnant or breastfeeding, had previous or current decompensated liver disease (including ascites, hepatic encephalopathy, or variceal hemorrhage), a liver transplant, cholangiocarcinoma, hepatocellular carcinoma, a Model for End-Stage Liver Disease score of >12 at screening (unless due to an alternate etiology such as therapeutic anticoagulation), or a Child–Pugh score of >6 at screening (unless due to an alternative etiology such as Gilbert syndrome or therapeutic anticoagulation). Full eligibility criteria are provided in Supplementary Digital Content (see Supplementary Table S1, http://links.lww.com/CTG/B163).

### Outcomes and assessments

The primary endpoint of the study was safety and tolerability assessed by treatment-emergent adverse events (TEAEs), clinical laboratory tests, and vital signs. TEAEs were summarized by system organ class and preferred terms using the MedDRA version 24.1. The Common Terminology Criteria for Adverse Events version 5.0 was used to assign toxicity grades (0–4) to laboratory results for analysis.

Exploratory endpoints included liver biochemistry, tests of liver function, fibrosis markers, PD biomarkers of bile acid homeostasis, HRQoL, and pruritus. Liver biochemistry and function were measured by changes from baseline in serum concentrations of alkaline phosphatase (ALP), γ-glutamyl transferase (GGT), ALT, aspartate aminotransferase (AST), total bilirubin, direct albumin, and INR. Fibrosis markers were assessed by changes from baseline in liver stiffness by FibroScan and ELF score, which included tissue inhibitor of matrix metalloproteinase 1 (TIMP1), procollagen type 3 N-terminal propeptide, and hyaluronic acid. PD biomarkers of bile acid homeostasis were measured by changes from baseline in plasma concentrations of fasting FGF19 (enzyme-linked immunosorbent assay, Q2 Solutions), fasting C4 (mass spectrometry; Metabolon), serum fasting cholic acid (mass spectrometry; Metabolon), and serum fasting total bile acids (enzymatic reaction assay; Covance by Labcorp). Blood samples were collected after 8 hours of fasting and before study drug administration at baseline (day 1); weeks 1, 4, 8, 12; and follow-up (week 16). Time point PD samples were collected at 2 and 4 hours after each dose at weeks 4, 8, and 12. HRQoL was evaluated by changes from baseline in the Chronic Liver Disease Questionnaire, EuroQol five dimensions questionnaire (EQ-5D), and Short Inflammatory Bowel Disease Questionnaire (for patients with a history of IBD), which were completed at baseline and week 12. Pruritus was assessed by changes from baseline in the pruritus Visual Analog Scale (VAS) and 5D itch scale at weeks 1, 4, 8, and 12.

### Statistical analyses

Owing to the exploratory nature of this study, no formal power calculations were used to determine sample size. Safety and efficacy outcomes were summarized and analyzed based on the safety analysis set, which included all patients who received at least one dose of the study drug. For continuous efficacy and HRQoL outcomes, descriptive statistics (n, interquartile range [IQR]) were calculated for baseline and postbaseline visits and for absolute changes and percentage changes from baseline at postbaseline visits. For categorical variables, descriptive statistics were calculated with the number and percentage of participants in each category. No formal statistical comparisons were performed for most efficacy outcomes, which were evaluated for exploratory purposes. A repeated measures mixed-effect analysis of variance was used to model longitudinal log-transformed values of PD markers of bile acid homeostasis (FGF19, C4 cholic acid, and total bile acids) with fixed effect terms for visit, postdose hour, and their interactions and patient as a random effect. Point estimates and 95% confidence intervals (95% CIs) were calculated. Owing to the small number of observations, more complex models were not used because of issues with convergence.

Values for missing data were not imputed. Missing baseline safety laboratory data and vital signs were, however, replaced with a screening result if available. If no pretreatment laboratory values were available, the baseline values were assumed to be normal (i.e., no grade) for the summary of graded laboratory abnormalities.

## RESULTS

### Study population

**Figure 1.**
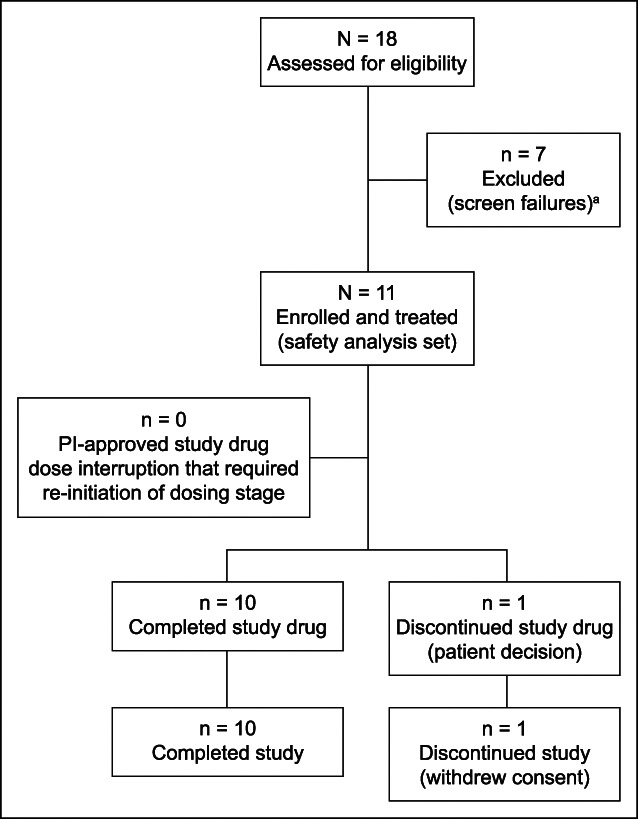
Participant flow. ^a^Detailed reasons for screen failures are provided in Supplementary Digital Content (see Supplementary Table 2, http://links.lww.com/CTG/B163). PI, principal investigator.

Patients were enrolled in the study between October 17, 2019, and June 8, 2021. Enrollment was terminated early by the sponsor on July 30, 2021, owing to challenges with recruitment and the impact of the COVID-19 pandemic on conducting clinical research. Patient disposition is presented in Figure 1. Of the 18 patients who were screened, 7 were screen failures (full details are provided in Supplementary Digital Content [see Supplementary Table S2, http://links.lww.com/CTG/B163]) and 11 were enrolled in this study. One patient discontinued the study drug before the cilofexor dose was escalated to 100 mg and subsequently discontinued the study. All other patients received treatment with escalating doses of cilofexor (30, 60, and 100 mg QD) over 12 weeks. One patient had temporary treatment interruption for 2 days during the 60 mg dosing period owing to peripheral edema but later completed 100 mg dosing. There were no PI-approved study drug interruptions of >5 days that required reinitiation of the 4-week dosing stage. Most patients (10/11 [90.9%]) were at least 90% compliant with the study drug.

Patient demographics and baseline characteristics are presented in Table 1. Similar proportions of men and women were included in the study, most of whom (10/11 [90.9%]) were White. The median (IQR) age was 48 (37–56) years, over half (6/11 [54.5%]) of the patients had a history of IBD, and one patient had small duct PSC. Median (IQR) baseline liver biochemistry levels were 510 U/L (269–592) for ALP, 344 U/L (208–455) for GGT, 56 U/L (37–89) for ALT, and 69 U/L (37–86) for AST. Median (IQR) baseline total bilirubin was 1.1 mg/dL (0.8–1.5). No patients were treated concomitantly with fibrates during the study.

**Table 1.** Patient demographics and baseline characteristics (safety analysis set)

Patient characteristic	Cilofexor (N = 11)
Age, yr	48 (37–56)
Sex, male	6 (55)
Race, White	10 (91)
IBD	6 (55)
UDCA therapy	5 (45)
UDCA, mg	900 (700–1,200)
History of pruritus	4 (36)
Platelets, ×10^3^/μL	201 (113–218)
Liver biochemistry markers	
ALP, U/L	510 (269–592)
GGT, U/L	344 (208–455)
ALT, U/L	56 (37–89)
AST, U/L	69 (37–86)
Liver function markers	
Total bilirubin, mg/dL	1.1 (0.8–1.5)
Liver fibrosis markers	
ELF score	10.4 (9.5–12.6)
Transient elastography, kPa	18.0 (8.5–67.6)
PD markers of bile acid homeostasis	
Fasting total bile acids, µM	25.3 (11.7–52.9)

Data shown as median (IQR) or n (%).

ALP, alkaline phosphatase; ALT, alanine aminotransferase; AST, aspartate aminotransferase; ELF, enhanced liver fibrosis; GGT, γ-glutamyl transferase; IBD, inflammatory bowel disease; IQR, interquartile range; PD, pharmacodynamic; UDCA, ursodeoxycholic acid.

### Safety outcomes

All patients received the study drug for at least 4 weeks, and most patients (9/11 [81.8%]) received it for 12 weeks. Median (IQR) exposure to cilofexor was 12.1 (12.0–12.3) weeks.

An overall summary of TEAEs and laboratory abnormalities is presented in Table 2. There were no deaths, serious TEAEs, or TEAEs leading to permanent study drug discontinuation; however, one patient with grade 2 peripheral edema had a brief interruption of cilofexor treatment for 2 days during the 60 mg dosing. No cases of ascending cholangitis, ascites, hepatic decompensation, hepatic encephalopathy, liver transplantation (or qualification for liver translation), portal hypertension-related upper gastrointestinal bleeding, or diagnoses of cholangiocarcinoma or hepatocellular carcinoma occurred during the study. Nine participants (81.8%) had at least one TEAE, most of which were grade 1 (4/11 [36.4%]) or grade 2 (3/11 [27.3%]) in severity. TEAEs occurring in at least 10% of patients were pruritus (8/11 [72.7%]), fatigue, headache, nausea, and upper respiratory tract infection (2/11 [18.2%] each). TEAEs of grade 3 or higher were reported in 2 patients (18.2%) and included one grade 3 TEAE of pruritus and one grade 3 TEAE of jaw fracture, lip injury, and skin laceration.

**Table 2.** Overall summary of TEAEs and laboratory abnormalities (safety analysis set)

TEAEs	Cilofexor (N = 11)
Any TEAE	9 (81.8)
Grade 2 or higher	5 (45.5)
TEAEs related to study drug	9 (81.8)
Serious TEAEs	0
TEAEs leading to discontinuation	0
Deaths	0
TEAEs seen in ≥10% of participants	
Pruritus	8 (72.7)
Fatigue	2 (18.2)
Headache	2 (18.2)
Nausea	2 (18.2)
Upper respiratory tract infection	2 (18.2)
Laboratory abnormalities	11 (100.0)
Grade 1	6 (54.5)
Grade 2	4 (36.4)
Grade 3	1 (9.1)
Grade 4	0

Data shown as n (%).

TEAE, treatment-emergent adverse event.

Nine patients (81.8%) had at least 1 TEAE considered related to the study drug, which included pruritus (7/11 [63.6%]), headache, nausea (2/11 [18.2%] each), peripheral edema, fatigue, and defecation urgency/diarrhea (1/11 [9.1%] each). The grade 3 TEAE of pruritus was considered related to study drug but was not classified as a serious TEAE. All participants had at least 1 laboratory abnormality, most of which were grade 1 (6/11 [54.5%]) or grade 2 (4/11 [36.4%]) in severity. One patient (9.1%) had grade 3 anemia.

### Efficacy outcomes

#### Liver biochemistry and function markers.

**Figure 2.**
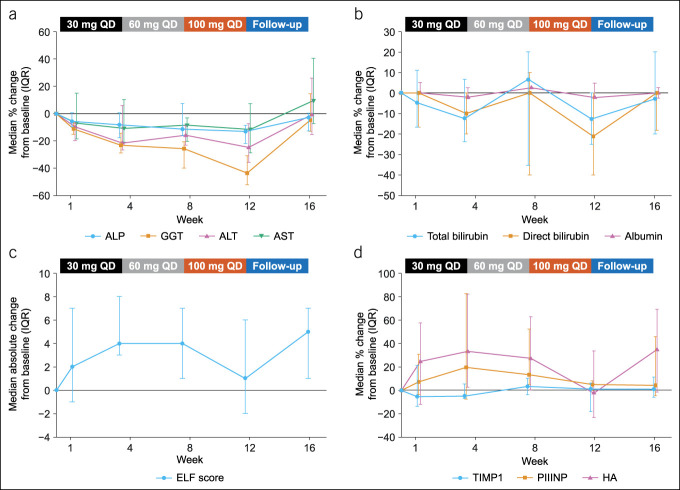
Longitudinal changes from baseline to follow-up (week 16) in efficacy outcomes. (**a**) Median percentage change (IQR) in liver biochemistry markers, (**b**) median percentage change (IQR) in liver function markers, (**c**) median absolute change (IQR) in ELF score, and (**d**) median percentage change (IQR) in fibrosis markers TIMP1, PIIINP, and HA. ALP, alkaline phosphatase; ALT, alanine aminotransferase; AST, aspartate aminotransferase; ELF, enhanced liver fibrosis; GGT, γ-glutamyl transferase; HA, hyaluronic acid; IQR, interquartile range; PIIINP, procollagen type 3 N-terminal propeptide; QD, daily; TIMP1, tissue inhibitor of matrix metalloproteinase 1.

Liver enzyme concentrations showed a decreasing trend from baseline to week 12, with a marked reduction in GGT. Concentrations reverted to near baseline levels during the 4-week follow-up period (Table 3 and Figure 2a). Median percentage changes (IQR) from baseline to week 12 were −13.0% (−21.9 to −8.6) for ALP, −43.5% (−52.1 to −30.8) for GGT, −24.8% (−35.7 to −7.4) for ALT, and −11.6% (−28.7 to 7.3) for AST. After 12 weeks of cilofexor treatment, all markers of liver function except INR showed minor reductions from baseline, reverting to near baseline levels in the 4-week follow-up period (Table 3 and Figure 2b). Median percentage changes (IQR) from baseline to week 12 were −12.7% (−25.0 to 0.0) for total bilirubin, −21.2% (−40.0 to 0.0) for direct bilirubin, −2.3% (−2.8 to 4.8) for serum albumin, and 0.0% (0.0 to 10.0) for INR.

**Table 3.** Absolute changes from baseline in liver biochemistry, liver function tests, noninvasive fibrosis biomarkers, and PD markers of bile acid homeostasis at week 12 (safety analysis set)

Parameter	Cilofexor (N = 11)
Liver biochemistry markers	
ALP, U/L	−62 (−88 to −11)
GGT, U/L	−147 (−288 to −104)
ALT, U/L	−16 (−20 to −2)
AST, U/L	−4 (−13 to 7)
Liver function markers	
Total bilirubin, mg/dL	−0.1 (−0.4 to 0.0)
Direct bilirubin, mg/dL	−0.1 (−0.2 to 0.0)
Albumin, g/dL	−0.1 (−0.1 to 0.2)
INR	0.0 (0.0 to 0.1)
Liver fibrosis markers	
Transient elastography, kPa	0.2 (−2.1 to 2.6)
ELF score	0.1 (−0.2 to 0.6)
TIMP1, ng/mL	3.0 (−58.6 to 22.1)
PIIINP, ng/mL	0.9 (0.0 to 1.9)
Hyaluronic acid, ng/mL	4.5 (−23.7 to 48.8)
PD markers of bile acid homeostasis	
Fasting FGF19, pg/mL	−13.4 (−30.5 to 25.6)
Fasting C4, ng/mL	−2.600 (−6.320 to −1.882)
Fasting total bile acids, µM	−3.4 (−5.6 to 3.0)
Fasting cholic acid, ng/mL	−9.11 (−53.68 to −1.34)

Data shown as median (IQR).

ALP, alkaline phosphatase; ALT, alanine aminotransferase; AST, aspartate aminotransferase; C4, 7α-hydroxy-4-cholesten-3-one; ELF, enhanced liver fibrosis; FGF19, fibroblast growth factor 19; GGT, γ-glutamyl transferase; INR, international normalized ratio; IQR, interquartile range; PD, pharmacodynamic; PIIINP, procollagen type 3 N-terminal propeptide; TIMP1, tissue inhibitor of matrix metalloproteinase 1.

#### Liver fibrosis markers.

Except for TIMP1, noninvasive liver fibrosis markers had generally increased from baseline to weeks 4 and 8 (30–60 mg QD dosing period), before declining to near baseline levels at week 12 (100 mg QD dosing period) (Table 3, Figure 2c,d). Median absolute change (IQR) in ELF score was +0.4 (0.1–0.7) from baseline to week 8 and +0.1 (−0.2 to 0.6) from baseline to week 12. Median absolute change (IQR) in liver stiffness from baseline to week 12 was +0.2 kPa (−2.1 to 2.6). Median percentage changes (IQR) from baseline to week 12 were +1.2% (−18.1 to 6.3) for TIMP1, +5.0% (0.0–8.3) for procollagen type 3 N-terminal propeptide, and −2.4% (−23.1 to 33.7) for hyaluronic acid levels (Table 3 and Figure 2d). ELF scores increased during the 4-week follow-up period after the last dose was received (Figure 2c), with similar changes in hyaluronic acid levels observed during this period (Figure 2d).

#### PD markers of bile acid homeostasis.

**Figure 3.**
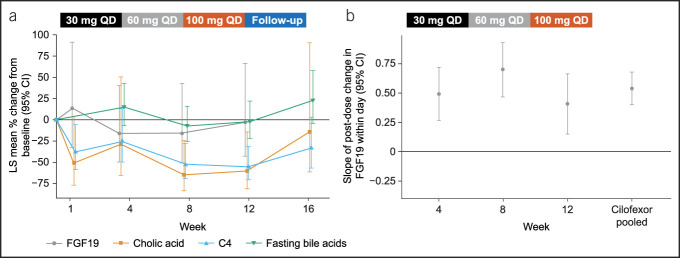
Changes in PD markers of bile acid homeostasis with cilofexor treatment. (**a**) Longitudinal percentage changes in levels of fasting plasma FGF19, C4, cholic acid, and total bile acids at trough from baseline to follow-up (week 16). The decrease in C4 and cholic acid biomarkers was statistically significant at weeks 8 and 12 as their corresponding 95% CIs did not include zero. (**b**) Slopes of changes of FGF19 levels at each visit and for pooled doses/visits. The 95% CIs of the FGF19 slopes do not include zero, thus signifying their statistical significance. C4, 7α-hydroxy-4-cholesten-3-one; CI, confidence interval; FGF19, fibroblast growth factor 19; LS, least squares; PD, pharmacodynamic.

Cilofexor significantly decreased serum levels of C4 and cholic acid at trough at weeks 8 and 12 (Figure 3a and Table 3). Least-squares mean percentage changes (95% CI) in C4 from baseline to weeks 4, 8, and 12 were −24.7% (−50.0 to 13.4), −52.3% (−68.8 to −27.2), and −55.3% (−70.8 to −31.6), respectively. Least-squares mean percentage changes (95% CI) in cholic acid from baseline to weeks 4, 8, and 12 were −28.6% (−66.1 to 50.6), −64.9% (−83.7 to −24.3), and −60.5% (−81.8 to −14.2), respectively.

Although there were no significant changes in FGF19 fasting plasma levels at trough at any visits (Figure 3a), there were significant transient increases in FGF19 plasma levels at 2 and 4 hours after cilofexor administration (Figure 3b, see Supplementary Figure 1a, http://links.lww.com/CTG/B163). By contrast, serum C4 remained at similar levels at 2 and 4 hours after cilofexor administration (see Supplementary Figure 1b, http://links.lww.com/CTG/B163).

There were no significant changes in fasting total bile acids at trough at any visits (Figure 3a), nor were there any significant increases 2 or 4 hours postdose (data not shown).

#### HRQoL and pruritus measures.

Changes in HRQoL (Chronic Liver Disease Questionnaire, EQ-5D, and Short Inflammatory Bowel Disease Questionnaire) scores from baseline to week 12 were minimal (Table 4). There were no clinically relevant changes from baseline to week 12 in median pruritus VAS response or 5D itch total scores (Table 5). The 8 patients who experienced a TEAE of pruritus had higher median (IQR) baseline VAS and 5D itch total scores (12 [0–27] and 12 [10–13], respectively) than those patients who did not experience pruritus (1 [0–3] and 7 [5–9], respectively). In the 6 patients with IBD, there was minimal change in IBD symptom severity from baseline to week 12 (data not shown). No rectal bleeding was reported during the treatment period; however, 1 patient experienced rare, minimal rectal bleeding at follow-up (week 16). Three patients reported no change in stool frequency throughout the study. Two patients reported a daily bowel movement of 3–5 stools more than on a typical day once during the study (one at week 8 and one at week 12) and <3 more stools than on a typical day at follow-up (both at week 16). A third patient had <3 more stools than on a typical day at week 4.

**Table 4.** HRQoL outcomes assessed by CLDQ, EQ-5D, and SIBDQ (safety analysis set)

	Baseline	Week 12	Change from baseline
CLDQ score	n = 11	n = 10	n = 10
Abdominal symptoms	5.3 (4.7 to 7.0)	6.2 (4.7 to 7.0)	0.0 (−0.3 to 0.3)
Fatigue	5.0 (3.4 to 6.2)	5.7 (4.8 to 6.8)	0.5 (0.0 to 0.6)
Systemic symptoms	5.6 (4.6 to 6.6)	5.8 (4.8 to 6.6)	0.2 (0.0 to 0.8)
Activity	7.0 (5.0 to 7.0)	7.0 (5.0 to 7.0)	0.0 (0.0 to 0.0)
Emotional function	6.4 (4.9 to 6.9)	6.3 (5.4 to 6.9)	0.0 (−0.4 to 0.4)
Worry	5.0 (4.6 to 7.0)	6.4 (4.8 to 7.0)	0.0 (−0.2 to 0.8)
Overall score	5.7 (4.7 to 6.5)	6.1 (5.4 to 6.7)	0.1 (0.0 to 0.3)
EQ-5D score	n = 11	n = 10	n = 10
Mobility	1 (1 to 1)	1 (1 to 1)	0 (0 to 0)
Self-care	1 (1 to 1)	1 (1 to 1)	0 (0 to 0)
Usual activities	1 (1 to 2)	1 (1 to 1)	0 (0 to 0)
Pain/discomfort	1 (1 to 2)	1 (1 to 2)	0 (0 to 0)
Anxiety/depression	1 (1 to 2)	1 (1 to 2)	0 (0 to 0)
SIBDQ score	n = 6	n = 5	n = 5
	63 (53 to 66)	63 (55 to 66)	0 (−5 to 4)

Data shown as median (IQR).

The safety analysis set was used to assess CLDQ and EQ-5D. SIBDQ was assessed in patients with a history of IBD. Data from the patient with early termination were not included in these assessments.

CLDQ, Chronic Liver Disease Questionnaire; EQ-5D, EuroQol five dimensions questionnaire; HRQoL, health-related quality of life; IBD, inflammatory bowel disease; IQR, interquartile range; SIBDQ, Short Inflammatory Bowel Disease Questionnaire.

**Table 5.** Change from baseline in pruritus assessed by VAS and 5D itch (safety analysis set)

Change from baseline	VAS		5D itch total	
Week 1	n = 11	2 (0 to 11)	n = 10	1 (0 to 1)
Week 4	n = 11	0 (−9 to 2)	n = 10	0 (−2 to 2)
Week 8	n = 10	0 (−16 to 5)	n = 9	0 (−1 to 0)
Week 12	n = 10	0 (0 to 4)	n = 9	0 (−1 to 2)
Week 16 (follow-up)	n = 10	0 (−25 to 4)	n = 9	0 (−2 to 2)

Data shown as median (IQR).

IQR, interquartile range; VAS, Visual Analog Scale.

## DISCUSSION

Treatment with cilofexor 30 mg QD (weeks 1–4), 60 mg QD (weeks 4–8), and 100 mg QD (weeks 8–12) was well tolerated and improved markers of cholestasis in patients with compensated cirrhosis due to PSC. The safety profile was consistent with that previously reported in patients with PSC without cirrhosis (13), and there were no deaths, serious TEAEs, or TEAEs leading to permanent study drug discontinuation. Furthermore, no participants had hepatic decompensation or other liver-related events or biliary complications such as ascending cholangitis.

Reductions in GGT and PD markers of bile acid homeostasis were observed from baseline to week 12. In contrast to the reductions in liver biochemistry markers previously observed in patients with noncirrhotic PSC after 12 weeks of cilofexor treatment (13), no clinically meaningful changes in concentrations of ALP, ALT, or AST were observed in this study. This may be explained by the presence of more advanced disease in patients with compensated cirrhosis due to PSC. Changes observed in liver fibrosis markers were within measurement variability of the tests. Thus, the relatively small variations in ELF score during the short duration of the trial should not be overinterpreted.

Based on the drug's mechanism of action as an FXR agonist, FGF19 levels would be expected to transiently increase with cilofexor treatment. Indeed, previous data from patients with PSC showed an elevated plasma FGF19 response for up to 8 hours post-treatment with chenodeoxycholic acid, a natural FXR agonist (20). In this study, a significant transient increase in FGF19 levels was observed with cilofexor within hours of administration. These findings are similar to those from a previous trial in patients with PSC without cirrhosis who were treated with 100 mg cilofexor QD (13) and in a study with healthy volunteers treated with 10, 30, 100, or 300 mg cilofexor QD (21), in which rapid, dose-dependent increases in FGF19 levels after administration of cilofexor were observed. Furthermore, the decreases in C4 and cholic acid levels in this study confirm reduced bile acid synthesis (22), which is driven by the increase in FGF19 levels and confirms the PD mechanism of cilofexor.

Pruritus is a common side effect associated with FXR agonists used to treat metabolic dysfunction-associated steatotic liver disease and cholestatic liver diseases (23). In addition, liver cirrhosis is more prevalent in patients with pruritus than those without and may increase the severity of pruritus (24,25). In this study, 7 patients reported pruritus that was considered related to cilofexor by the PI. One event was grade 3 in severity but not considered serious. According to data from a systematic review, the severity of pruritus is positively correlated with impaired HRQoL (26). Despite the high frequency of pruritus in this study, there were no clinically relevant changes from baseline to week 12 in VAS and 5D itch pruritus assessments or general HRQoL measures. These results indicate that the pruritus TEAEs did not substantially add to the patient burden. The apparent discordance between the high frequency of pruritus and lack of change in pruritus or HRQoL measures may be explained by the higher baseline scores of pruritus measures in patients who experienced a TEAE of pruritus than in those who did not; however, these results should be interpreted with caution owing to the small sample size. It may also be because pruritus was recorded as a TEAE even if patients had experienced it before commencing treatment with cilofexor (i.e., the pruritis event was not necessarily a worsening from baseline) and that this study permitted the management of pruritus and included a formal management plan for patients who developed significant pruritus.

Effective pharmacological therapy to halt the progression of PSC remains an unmet need. Liver transplantation is an option for patients with a qualifying Model for End-Stage Liver Disease score (27); however, specific challenges can arise for patients with PSC who undergo liver transplantation, including management of colitis, graft selection, anticoagulation, cellular rejection, and disease recurrence (28). Five of the 11 patients in this study were on UDCA therapy at the start of the study. Early clinical studies showed that UDCA can reduce levels of biochemical markers of cholestasis and reduce PSC disease activity (29,30), but no improvement in survival has been demonstrated (31,32). Data from this exploratory study suggest that further evaluation of cilofexor for the treatment of patients with compensated cirrhosis due to PSC may be warranted.

This study has several limitations, most notably its small sample size which was compounded by recruitment issues. Moreover, the study lacked a placebo control group and was short in duration, limiting any comparative assessment of the impact of cilofexor on clinical outcomes in patients with cirrhosis due to PSC. Nevertheless, a strength of this study was the inclusion of patients with cirrhosis—a population that is typically excluded from clinical trials despite their high unmet medical need.

In conclusion, this study showed that escalating doses of cilofexor over 12 weeks were well tolerated and improved markers of cholestasis in patients with compensated cirrhosis due to PSC.

## CONFLICTS OF INTEREST

**Guarantor of the article:** William T. Barchuk, MD.

**Specific author contributions:** All authors made substantial contributions to the intellectual content of the paper and approved the final version of the manuscript for publication. C.L., J.X., A.N.B., X.Liu., X.Lu, C.C., R.P.M., and K.V.K.: conception and design. C.L., S.C., P.M., V.L., C.S.L., J.H., E.M., R.M., K.R., and K.V.K.: acquisition of data. C.L., J.X., V.A.M., X.Liu, X.Lu, W.T.B., T.R.W., C.C., R.P.M., and K.V.K.: analysis and interpretation of data. All authors: critical revision of the manuscript for intellectual content.

**Financial support:** Funding for the study and writing support was provided by Gilead Sciences, Inc.

**Potential competing interests:** C.L. has received research grants from Calliditas Therapeutics, Cara Therapeutics, CymaBay Therapeutics, GENFIT, Gilead Sciences, Inc., GlaxoSmithKline, HighTide Therapeutics, Intercept Pharmaceuticals, Ipsen, Mirum Pharmaceuticals, Novartis, Target RWE, and Zydus Lifesciences; and has received consulting fees from Calliditas Therapeutics, CymaBay Therapeutics, Gilead Sciences, Inc., GlaxoSmithKline, Intercept Pharmaceuticals, Ipsen, Mirum Pharmaceuticals, and Target Real World Evidence. S.C. has received research grants from Avanos, Cour Pharmaceuticals, Durect, Galectin Therapeutics, GENFIT, Gilead Sciences, Inc., Inventiva, Madrigal Pharmaceuticals, and Zydus Lifesciences. P.M. has received research grants from Gilead Sciences, Inc. V.L. has received research grants from Akero Therapeutics, Albireo, AstraZeneca, Bristol Myers Squibb, CymaBay Therapeutics, GENFIT, Gilead Sciences, Inc., Hanmi Pharmaceutical, Ipsen, Intercept Pharmaceuticals, Inventiva, Madrigal Pharmaceuticals, Merck Sharp & Dohme, Novartis, Novo Nordisk, Perspectum, Pfizer, Pliant, Salix Pharmaceuticals, Target RWE, and Zydus Lifesciences. C.S.L. has received research grants from Bristol Myers Squibb, CymaBay, GENFIT, Gilead Sciences, Inc., Intercept, NGM, Novartis, Novo Nordisk, Pfizer, Salix Pharmaceuticals, and Target RWE. E.M. has received speaker fees from AbbVie, Eisai Pharmaceuticals, GileadSciences, Inc., and Salix. K.V.K. has received consultancy fees from 89bio, CymaBay Therapeutics, Enanta Pharmaceuticals, GENFIT, Gilead Sciences, Inc., HighTide Therapeutics, Inipharm, Intercept Pharmaceuticals, Ipsen, Madrigal Pharmaceuticals, Mirum Pharmaceuticals, NGM, Pliant, Pfizer, and Zydus Lifesciences; research grants from 89bio, Boston Pharmaceuticals, Corcept Therapeutics, CymaBay Therapeutics, GENFIT, Gilead Sciences, Inc., GlaxoSmithKline, Hanmi Pharmaceutical, Intercept Pharmaceuticals, Ipsen, Janssen, Madrigal Pharmaceuticals, Mirum Pharmaceuticals, Novo Nordisk, NGM, Pfizer, Pliant, Terns Pharmaceuticals, Viking Therapeutics, and Zydus Lifesciences; speaker fees from AbbVie, Gilead Sciences, Inc., Intercept Pharmaceuticals; and stock options from Inipharm. J.X., V.A.M., A.N.B., X.Liu, X.Lu, W.T.B., and T.R.W. are employed by and shareholders in Gilead Sciences, Inc. C.C. is employed by Inipharm and a shareholder in Gilead Sciences, Inc. R.P.M. is a shareholder in Gilead Sciences, Inc. J.H., R.M., and K.R. have no conflicts of interest.

**ClinicalTrials.gov identifier:** NCT04060147.

**Study Highlights**

**WHAT IS KNOWN**

✓ Primary sclerosing cholangitis (PSC) is a rare progressive chronic liver disease.
✓ There are no effective medical interventions that delay progression of PSC.
✓ A phase 2 trial showed that cilofexor improves cholestasis and circulating markers of liver injury in noncirrhotic PSC.

**WHAT IS NEW HERE**

✓ This was a phase 1b trial in patients with PSC and compensated cirrhosis.
✓ Escalating doses of cilofexor were well tolerated in patients with PSC and compensated cirrhosis.
✓ Cilofexor demonstrated target engagement with farnesoid X receptor activity and improved markers of cholestasis in patients with compensated cirrhosis due to PSC.

## Supplementary Material
