# Supplementary material for: Cilofexor in Patients With Compensated Cirrhosis Due to Primary Sclerosing Cholangitis: An Open-Label Phase 1B Study
Source: Clin Transl Gastroenterol. 2024 Jul 1;15(8):e00744. doi: 10.14309/ctg.0000000000000744 (PMC11346858; doi:10.14309/ctg.0000000000000744)
Supplement: Supplementary file 1 [file ct9-15-e00744-s001.docx]

# Supplementary material

# Cilofexor in Patients with Compensated Cirrhosis Due to Primary Sclerosing Cholangitis: an Open-label Phase 1B Study

**Supplementary Table 1.** Full eligibility criteria of study population.

| **Inclusion criteria** | |
| --- | --- |
| 1 | Willing and able to give informed consent before any study specific procedure |
| 2 | Men and nonpregnant, nonlactating women aged 18–70 years (inclusive based on date of the screening visit) |
| 3 | Diagnosis of PSC based on cholangiogram (magnetic resonance cholangiopancreatography, endoscopic retrograde cholangiopancreatography, or percutaneous transhepatic cholangiogram), or liver biopsy |
| 4 | Patient has evidence of cirrhosis based on at least one of the three following criteria:   1. Historical liver biopsy that reveals Ludwig stage F4 fibrosis (or equivalent) 2. Abdominal imaging with features consistent with cirrhosis in the opinion of the PI (e.g., small nodular liver, splenomegaly, evidence of portosystemic collaterals, diffuse surface irregularity) 3. Any one of the following completed at the screening visit:   i. FibroScan^®^ ≥ 14.4 kPA  ii. ELF™ score ≥ 11.3  iii. FibroTest^®^ ≥ 0.75 |
| 5 | Patient has the following laboratory parameters at screening, as determined by the central laboratory:   1. eGFR > 60 mL/min, as calculated by the Cockcroft-Gault equation 2. ALT ≤ 5 × ULN 3. Total bilirubin ≤ 2 mg/dL, unless the subject is known to have Gilbert’s syndrome or hemolytic anemia 4. INR ≤ 1.4, unless due to therapeutic anticoagulation 5. Platelet count ≥ 75,000/μL. Patients with evidence of high-risk esophageal or gastric varices in the opinion of the investigator are excluded 6. Negative anti-mitochondrial antibody |
|  |  |
| 6 | For patients receiving UDCA, the dose of UDCA must have been stable for at least 6 months before screening and anticipated to remain stable throughout the study. For patients not receiving UDCA, no UDCA use for at least 6 months before screening |
| 7 | For patients being administered biologic treatments (e.g., anti-tumor necrosis factor or anti-integrin monoclonal antibodies), immunosuppressants, or systemic corticosteroids, the dose must have been stable for at least 3 months before screening and anticipated to remain stable throughout the study |
| 8 | Women of childbearing potential must have a negative serum pregnancy test at screening and a negative urine pregnancy test before the first dose of the study drug on the baseline/day 1 visit |
| 9 | Patients of childbearing potential who engage in heterosexual intercourse must agree to use specified method(s) of contraception |
| 10 | Patients must be able to comply with the dosing instructions for study drug administration and able to complete the study schedule of assessments |
| **Exclusion criteria** | |
| 1 | Current or prior history of any of the following:   1. Decompensated liver disease, including ascites, hepatic encephalopathy, or variceal hemorrhage 2. Liver transplantation 3. Cholangiocarcinoma or hepatocellular carcinoma. If a dominant structure has been identified, cholangiocarcinoma must be adequately treated |
| 2 | MELD score > 12 at screening, unless due to an alternate etiology such as therapeutic anticoagulation |
| 3 | Child–Pugh score > 6 at screening, unless due to an alternative etiology such as Gilbert’s syndrome or therapeutic anticoagulation |
| 4 | Presence of moderate-to-severe itch in the opinion of the PI at screening and baseline/day 1 |
| 5 | Ascending cholangitis within 30 days before screening |
| 6 | Presence of a percutaneous drain or biliary stent |
| 7 | Other causes of liver disease including IgG4-related sclerosing cholangitis; PSC-autoimmune hepatitis overlap syndrome; secondary sclerosing cholangitis; and viral, metabolic, alcoholic, and other autoimmune conditions. Patients with hepatic steatosis may be included if there is no evidence of nonalcoholic steatohepatitis in the opinion of the PI |
| 8 | Current moderate-to-severe active IBD (including ulcerative colitis, Crohn’s disease, and indeterminate colitis). Note: Patients with IBD who currently have an external ostomy bag and/or proctocolectomy are not subject to this exclusion criterion and need not undergo IBD Symptom Severity Assessment. |
| 9 | Current or prior history of any of the following:   1. Malignancy within 5 years of screening with the following exceptions: 2. Adequately treated carcinoma *in situ* of the cervix 3. Adequately treated basal or squamous cell cancer or other localized nonmelanoma skin cancer.   Patients under evaluation for possible malignancy are not eligible.   1. Unstable cardiovascular disease as defined by any of the following: 2. Unstable angina, myocardial infarction, coronary artery bypass graft surgery or coronary angioplasty in the 6 months before screening 3. Transient ischemic attack or cerebrovascular accident in the 6 months before screening 4. Symptomatic obstructive valvular heart disease or hypertrophic cardiomyopathy 5. Symptomatic congestive heart failure 6. Uncontrolled or recurrent ventricular tachycardia or other arrhythmia requiring an automatic implantable cardioverter defibrillator. Stable, controlled atrial fibrillation is allowed. 7. Hypercoagulable condition or venous or arterial thromboembolic disease. |
| 10 | HIV infection (HIV antibody and HIV ribonucleic acid positive) |
| 11 | HBV infection (hepatitis B surface antigen positive) |
| 12 | HCV infection (HCV antibody and HCV ribonucleic acid positive). Patients cured of HCV infection ≥ 2 years prior to screening are eligible. |
| 13 | Habitual alcohol consumption > 21 oz/week for males or > 14 oz/week for females (1 oz/30 mL of alcohol is present in one 12 oz/360 mL beer, one 4 oz/120 mL glass of wine, and a 1 oz/30 mL measure of 40% proof alcohol) |
| 14 | Use of antibiotics (e.g., vancomycin, metronidazole, minocycline) for the treatment of PSC within 60 days before screening. Antibiotic prophylaxis for ascending cholangitis is permitted if the condition is stable in the opinion of the investigator for at least 6 months prior to screening. |
| 15 | Use of any prohibited concomitant medications as described in the protocol |
| 16 | Positive urine screen for amphetamines, cocaine, or opiates (i.e., heroin, morphine) at screening. Patients on stable methadone or buprenorphine maintenance treatment for at least 6 months prior to screening may be included. Patients with a positive urine drug screen due to prescription opioid-based medication are eligible if the prescription and diagnosis are reviewed and approved by the investigator. |

ALT, alanine transaminase; eGFR, estimated glomerular filtration rate; ELF, Enhanced Liver Fibrosis test; HBV, hepatitis B virus; HCV, hepatitis C virus; HIV, human immunodeficiency virus; IBD, inflammatory bowel disease; IgG4, immunoglobulin G4; INR, international normalized ratio; MELD, Model for End-stage Liver Disease; PI, principal investigator; PSC, primary sclerosing cholangitis; UDCA, ursodeoxycholic acid; ULN, upper limit of normal.

**Supplementary Table 2.** Full details of screen failures

| **Reason** | **Frequency** |
| --- | --- |
| **Failed to meet the following inclusion criteria** | 6 |
| Patient has evidence of cirrhosis based on at least one of the three following criteria:   1. Historical liver biopsy that reveals Ludwig stage F4 fibrosis (or equivalent) 2. Abdominal imaging with features consistent with cirrhosis in the opinion of the PI (e.g., small nodular liver, splenomegaly, evidence of portosystemic collaterals, diffuse surface irregularity) 3. Any one of the following completed at the screening visit:    1. FibroScan^®^ ≥ 14.4 kPA    2. ELF™ score ≥ 11.3    3. FibroTest^®^ ≥ 0.75 | 1 |
| Patient has the following laboratory parameters at screening^a^, as determined by the central laboratory:   1. eGFR > 60 mL/min 2. ALT ≤ 5x ULN 3. total bilirubin ≤ 1.5x ULN (except in cases of Gilbert syndrome) 4. INR ≤ 1.4 5. platelets ≥ 100,000/mm^3^ 6. negative anti-mitochondrial antibody | 1 |
| Patient has the following laboratory parameters at screening, as determined by the central laboratory:   1. eGFR > 60 mL/min 2. ALT ≤ 5x ULN 3. total bilirubin ≤ 2mg/dL (except in cases of Gilbert syndrome) 4. INR ≤ 1.4 5. platelets ≥ 75,000/mm^3^ 6. negative anti-mitochondrial antibody | 2 |
| Patients must be able to comply with the dosing instructions for study drug administration and able to complete the study schedule of assessments | 1 |
| **Met the following exclusion criteria** | 3 |
| Ascending cholangitis within 30 days before screening | 1 |
| Presence of a percutaneous drain or biliary stent | 1 |
| Use of antibiotics (e.g., vancomycin, metronidazole, minocycline) for the treatment of PSC within 60 days before screening. | 1 |

The total frequency of reasons for screen failures (n = 9) is greater than the total number of patients with screen failures (n = 7) because there could be more than one reason per patient.

^a^Laboratory parameters differ to those for inclusion criterion 5 (Supplementary Table 1) owing to a protocol amendment.

ALT, alanine transaminase; eGFR, estimated glomerular filtration rate; INR, international normalized ratio; PSC, primary sclerosing cholangitis.

**Supplementary Figure 1.** Plasma/serum level changes in PD markers of bile acid homeostasis with cilofexor treatment for individual patients. Horizontal gray dashed lines show corresponding levels at baseline visit. Solid lines represent longitudinal changes within hours of cilofexor administration, predicted by a statistical model. **a)** fasting plasma FGF19, **b)** fasting serum C4.

C4, 7-alpha-hydroxy-4-cholesten-3-one; FGF19, fibroblast growth factor 19; PD, pharmacodynamic.
